# Hormonal Effects on Nodular GAVE

**DOI:** 10.4021/gr528w

**Published:** 2013-05-03

**Authors:** Alan Brijbassie, Abdullah Al Osaimi, Steven M Powell

**Affiliations:** a Virginia Tech Carilion School of Medicine, Roanoke, Virginia, USA; b Digestive Center of Excellence, University of Virginia Health System, Charlottesville, Virginia, USA

**Keywords:** Gastric antral vascular ectasia, Nodular antral gastropathy, Proton pump inhibitor

## Abstract

Gastric antral vascular ectasia (GAVE) and its nodular antral gastropathy (NAG) variant is a unique lesion associated with hypergastrinemic hormonal alterations that may be compounded by concurrent proton pump inhibitor (PPI) therapy. The use of octreotide as a somatostatin analogue and its role in the down regulation of variousenteric hormones has been well documented however its use in the management of NAG has not been widely reported. We herein present a case where octreotide induced gastrin down-regulation as well as PPI cessation facilitated NAG resolution.

## Introduction

Proton pump inhibitor (PPI) therapy can be very effective in the symptomatic and endoscopic remission of acid-peptic disorders. However, despite its increasingly widespread use; their side effect profile is just beginning to be unveiled. Here we present a case where hormonal disarray in conjunction with PPI therapy produced deleterious effects on nodular gastric antral vascular ectasia (GAVE). This case also highlights the importance of understanding the pathophysiologic basis of different disease states so as to provide comprehensive optimal therapeutic patient care.

## Case Report

A 49-year-old Caucasian male was referred from an outside facility for further evaluation of refractory iron deficiency anemia secondary to chronic intermittent melenic gastrointestinal bleeding. Despite multiple blood transfusions and iron supplementation, his anemia continued to worsen. Upper endoscopy performed at an outside facility described features of chronic gastritis hence proton pump inhibitor therapy was instituted. Despite this the frequency of melenic stools increased. Repeat upper endoscopy reported multiple friable antral polyps with histopathologic analysis reporting changes consistent with adenomatous tissue.

Our patient’s past medical history was notable for chronic active hepatitis C infection, coronary artery disease and chronic kidney disease. The patient was post pancreatic and renal transplantation. A liver biopsy in 2001 demonstrated mild fibrosis (grade 2, stage 1) consistent with chronic viral hepatitis C. Medications on referral to our institution included Nexium 40 mg daily, Iron sulfate 325 mg twice daily, ASA 81 mg, Carafate 1 g four times daily and cyclosporine 100 mg daily.

**Figure 1:**
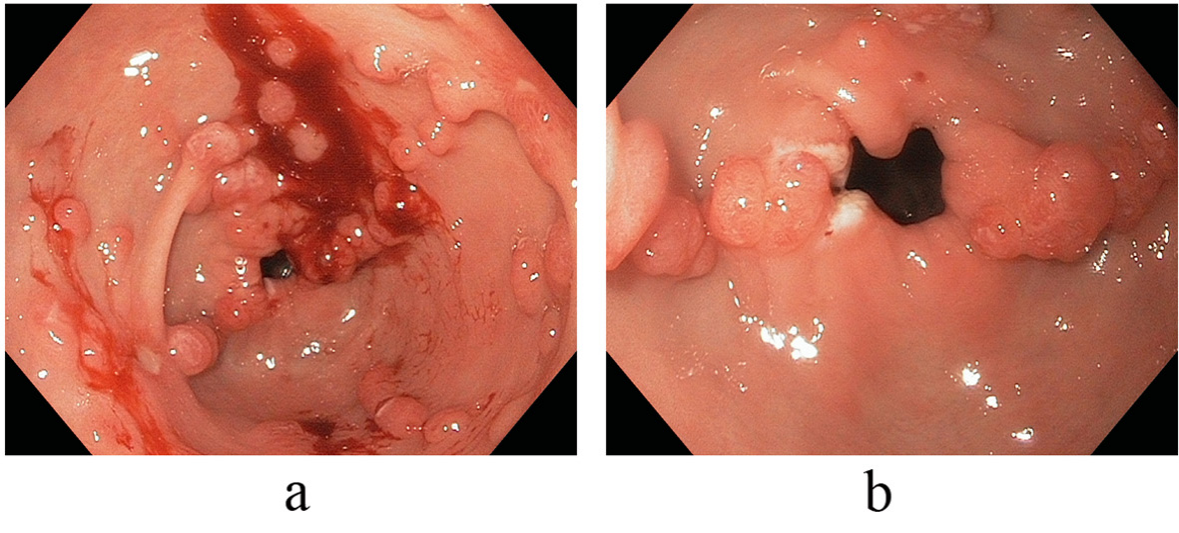
(a, b) Endoscopic view illustrative of multiple erythematous friable antral polyps and nodules ranging in size from 2 to 6 mm.

**Figure 2:**
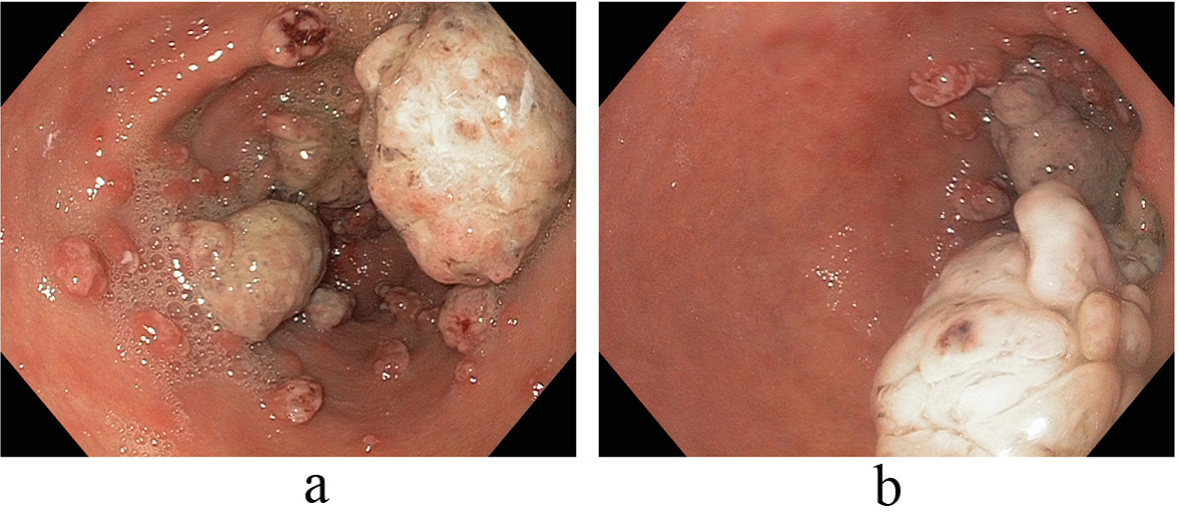
(a, b) Temporal increase in the size and associated friability of the nodular, polypoid lesions after APC (size increased from an average size of 6 mm at initial presentation to 20 mm after 8-months).

On referral to our facility, EGD evaluation revealed multiple erythematous friable antral polyps and nodules ranging in size from 2 to 6 mm (Fig. 1). Cautery treatments of these antral lesions were performed with Argon Plasma Coagulation (APC) and with snare cautery polypectomies of the larger lesions. Histopathologic review of these lesions and previous biopsies taken at the referring facility favored a diagnosis of hyperplastic/reactive polyps with no features of dysplasia. Moreover, vascular patterns of mucosal capillary dilation in the presence of foveolar-type epithelium consistent with gastric antral vascular ectasia (GAVE) were observed in these lesions. Additional investigations were performed to clarify other sources of gastrointestinal blood loss (enteroscopy, colonoscopy and capsule endoscopy) however were all negative with only lesions consistent with actively oozing nodular antral gastropathy (NAG; a nodular type of GAVE) left to account for his gastrointestinal bleeding and iron deficiency anemia. Pathologic reviews of biopsies for *Helicobacter pylori* were all negative and the patient’s PPI was adjusted to twice daily. Recurrent melenic gastrointestinal bleeding continued to occur despite multiple sessions of cautery with APC and snare polypectomies of the antral lesions. During the patient’s 12-month clinical course, four APC procedures were performed. Furthermore, serial EGD evaluations observed a temporal increase in the size and associated friability of the nodular, polypoid lesions after each cautery treatment. The size of the polypoid lesions increased from an average size of 6 mm at initial presentation to 20 mm after 8-months into his clinical course (Fig. 2).

**Figure 3:**
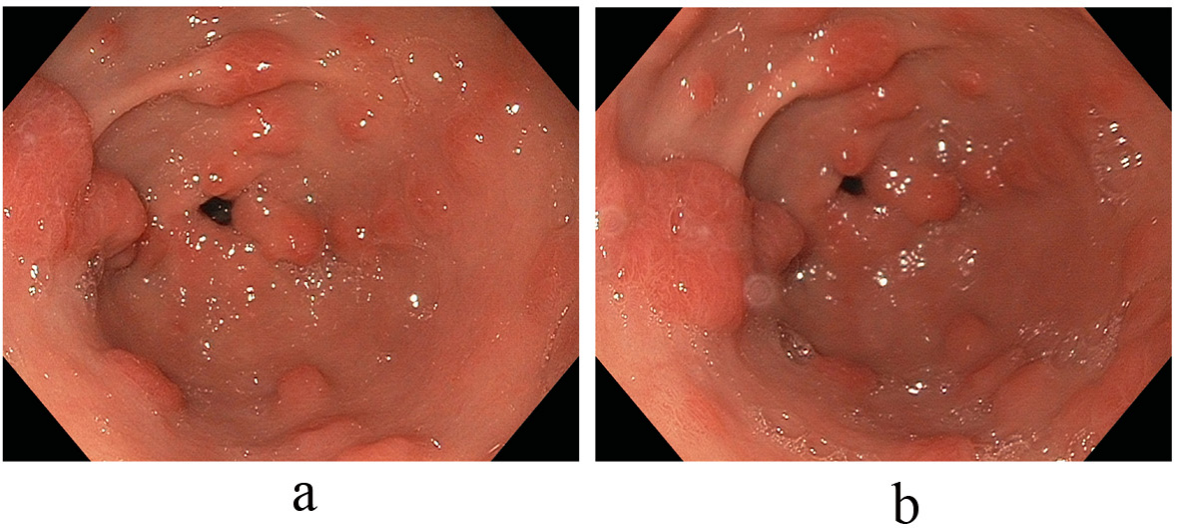
(a, b) Marked improvement in the antral vascular ectatic pattern observed since discontinuation of the PPI agent and initiation of the Octreotide therapy.

Anemia secondary to recurrent melenic stools continued to worsen with the patient requiring a total of 5 units of packed red blood cells in addition to his oral hematinic iron supplementation. These changes occurred on the background of chronic proton pump inhibitor therapy with the antral lesions reaching a size of 10 to 30 mm. Notably, an elevated serum gastrin level of 474 pg/mL (normal: 0 - 115 pg/mL) paralleled the growth of these polypoid antral lesions. With an increasing size of polypoid growth and continued recurrent melenic bleeding requiring periodic transfusions; additional strategies to combat these antral lesions were needed. We then decided to address the hormonal stimulation of these lesions and first discontinued the proton pump inhibitor agent and started an H2-blocker, Ranitidine 150 mg twice daily. An interval EGD noted stable antral lesions, but not significantly changed; thus additional hormonal treatment with Octreotide therapy was subsequently commenced with a dramatic effect on the antral lesions noted. Immediately after this intervention, a significant decrease in size and number of the antral lesions was observed, with only several 2 to 3 millimeter faintly erythematous nodules observed in the antrum without friability or oozing. In tandem, the levels of serum gastrin decreased exponentially as well with the initiation of Octreotide and the discontinuation of the PPI. Subsequent EGD studies confirmed marked improvements in the antral vascular ecstatic pattern observed since discontinuation of the PPI agent and initiation of the Octreotide therapy (Fig. 3). The patient’s ‘refractory’ anemia normalized over a 6-month period without the need for subsequent transfusions.

## Discussion

Gastric Antral Vascular Ectasia (GAVE) was first described in 1953 by Ryder et al [1]; it was only in 1984 that the term ‘watermelon stomach’ was coined by Jabarri et al whose endoscopic description of three patients presenting with gastric mucosal bleeding was noted for longitudinal antral folds convergent upon a pylorus containing visible columns of tortuous ectatic vessels resembling ‘spokes on a wheel’ [2].

The pathogenesis of the condition still remains an enigma; however two schools of thought are currently in vogue with the latter gaining more popularity. The first involves a predominantly mechanical component with the view that repeated prolapse of the antral mucosa by forceful peristalsis results in repeated obstruction of mucosal and submucosal blood flow leading to vascular ectasia; a view supported by the nearly equidistant and unique linear pattern of the blood vessels seen [2].

The second theory favors a biochemical origin borne out of the observation that the condition was more prevalent in patients with chronic liver disease. Recent studies have made a clear distinction between chronic liver disease as opposed to portal hypertension as the implicating factor. It was noted that GAVE lesions disappeared post liver transplant; however, portal pressure reduction strategies (porto-caval shunting or transjugular intra-hepatic portosystemic shunt or TIPS) had no effect [3-5]. It is postulated that the accumulation of vasoactive substances that are poorly metabolized by the cirrhotic liver results in angiogenesis and vasodilation. Gastrin, glucagon, nitric oxide, and TNF-alpha are mediators most commonly implicated in this setting [5]. This was further reinforced by the observation that the levels of these mediators are elevated in cirrhotic and are observed to be normal post liver transplant [5].

Treatment options for GAVE are diverse, however first line management is generally cautery given endoscopically such as with Argon Plasma Coagulation (APC) as it commonly provides resolution of bleeding, anemia and ablation of friable lesions [6, 7]. Nodular antral gastropathy (NAG) in co-existence with GAVE is rare though not unexpected as it has been shown that both conditions share similar biochemical features [8]. NAG was first reported by Takemoto et al in 1962; initially thought to be confined to young women, it has since been found in a wide range of adults [9]. Shimatani et al noted that serum gastrin levels were considerably higher in NAG patients than in control subjects with antral-predominant gastritis or pangastritis [8]. It was also noted that gastric acidity was significantly reduced in these NAG patients as measured by 24-hour intragastric pH-metry; a finding that could be explained by co-existent H. Pylori infection as well.

Gastrin is a linear peptide hormone produced by G-cells of the antrum and duodenum. Its effects include the stimulation of gastric blood flow, parietal cell maturation, increased antral muscle mobility with fundal growth and trophic effects on the GI tract. Reports of enterochromaffin cell hyperplasia and carcinoid tumors in hypergastrinemia settings have raised a safety concern in humans [10, 11]. Its secretion is stimulated by a variety of conditions including stomach distension, vagal stimulation, and hypercalcemia; however, achlorhydria/hypochlorhydria appears to be the most common reason. Chronic acid suppression with proton pump inhibitor (PPI) therapy has been well known to induce hypochlorhydria with secondary hypergastrinemia resulting in the aforementioned effects. Furthermore, hypergastrinemia has been linked to the development of Fundic Gland Polyps (FGP’s) [12, 13]. Treatment options for hypergastrinemic states include discontinuation of any inciting factors (for example, PPI therapy), and the use of Octreotide to downregulate its levels. Octreotide is a somatostatin analogue that has well characterized effects to negatively regulate a variety of hormones including the inhibition of hormonal release of serotonin, vasoactive intestinal peptide (VIP), secretin, motilin, pancreatic polypeptide, thyroid stimulating hormone and most notably gastrin. Octreotide has been demonstrated to decrease splanchnic blood flow which has been therapeutically beneficial in certain clinical circumstances.

### Conclusion

Gastric antral vascular ectasia and its NAG variant is a unique lesion associated with hormonal alterations that largely remain to be characterized. It has been demonstrated to be associated with a hypergastrinemia and when compounded by chronic proton pump inhibitor therapy which itself can lead to elevated gastrin, the ensuing ‘gastrinemic state’ may lead to untoward effects as evidenced in this case. Our case illustrates an instance where proton pump inhibitor therapy may have actually had deleterious effects. It also demonstrates the expanding use of Octreotide and the importance of understanding disease pathophysiology at a pathophysiologic level in order to provide optimal comprehensive therapeutic care.
